# Selepressin, a novel selective vasopressin V_1A_ agonist, is an effective substitute for norepinephrine in a phase IIa randomized, placebo-controlled trial in septic shock patients

**DOI:** 10.1186/s13054-017-1798-7

**Published:** 2017-08-15

**Authors:** James A. Russell, Jean-Louis Vincent, Anne Louise Kjølbye, Håkan Olsson, Allan Blemings, Herbert Spapen, Peder Carl, Pierre-Francois Laterre, Lars Grundemar

**Affiliations:** 1 Centre for Heart Lung Innovation, Division of Critical Care Medicine, St. Paul’s Hospital, University of British Columbia, 1081 Burrard Street, Vancouver, BC V6Z 1Y6 Canada; 2 Department of Intensive Care, ULB Erasme University Hospital (Université Libre de Bruxelles), Route de Lennik 808, B-1070 Brussels, Belgium; 3 Ferring Pharmaceuticals A/S, Kay Fiskers Plads 11, DK-2300 Copenhagen, Denmark; 4 Dienst Intensieve Geneeskunde, UZ Brussel, Laarbeeklaan 101, B-1090 Brussels, Belgium; 5 Anaestesiology Department, Hvidovre Hospital, DK-2650 Hvidovre, Denmark; 6 Clinique Universitaire St.-Luc, Service des Soins Intensifs, 10 Avenue Hippocrate, B-1200 Brussels, Belgium

**Keywords:** Selepressin, V_1A_ agonist, Norepinephrine, Mechanical ventilation, Fluid balance, Septic shock

## Abstract

**Background:**

Vasopressin is widely used for vasopressor support in septic shock patients, but experimental evidence suggests that selective V_1A_ agonists are superior. The initial pharmacodynamic effects, pharmacokinetics, and safety of selepressin, a novel V_1A_-selective vasopressin analogue, was examined in a phase IIa trial in septic shock patients.

**Methods:**

This was a randomized, double-blind, placebo-controlled multicenter trial in 53 patients in early septic shock (aged ≥18 years, fluid resuscitation, requiring vasopressor support) who received selepressin 1.25 ng/kg/minute (*n* = 10), 2.5 ng/kg/minute (*n* = 19), 3.75 ng/kg/minute (*n* = 2), or placebo (*n* = 21) until shock resolution or a maximum of 7 days. If mean arterial pressure (MAP) ≥65 mmHg was not maintained, open-label norepinephrine was added. Co-primary endpoints were maintenance of MAP >60 mmHg without norepinephrine, norepinephrine dose, and proportion of patients maintaining MAP >60 mmHg with or without norepinephrine over 7 days. Secondary endpoints included cumulative fluid balance, organ dysfunction, pharmacokinetics, and safety.

**Results:**

A higher proportion of the patients receiving 2.5 ng/kg/minute selepressin maintained MAP >60 mmHg without norepinephrine (about 50% and 70% at 12 and 24 h, respectively) vs. 1.25 ng/kg/minute selepressin and placebo (*p* < 0.01). The 7-day cumulative doses of norepinephrine were 761, 659, and 249 μg/kg (placebo 1.25 ng/kg/minute and 2.5 ng/kg/minute, respectively; 2.5 ng/kg/minute vs. placebo; *p* < 0.01). Norepinephrine infusion was weaned more rapidly in selepressin 2.5 ng/kg/minute vs. placebo (0.04 vs. 0.18 μg/kg/minute at 24 h, *p* < 0.001), successfully maintaining target MAP and reducing norepinephrine dose vs. placebo (first 24 h, *p* < 0.001). Cumulative net fluid balance was lower from day 5 onward in the selepressin 2.5 ng/kg/minute group vs. placebo (*p* < 0.05). The selepressin 2.5 ng/kg/minute group had a greater proportion of days alive and free of ventilation vs. placebo (*p* < 0.02). Selepressin (2.5 ng/kg/minute) was well tolerated, with a similar frequency of treatment-emergent adverse events for selepressin 2.5 ng/kg/minute and placebo. Two patients were infused at 3.75 ng/kg/minute, one of whom had the study drug infusion discontinued for possible safety reasons, with subsequent discontinuation of this dose group.

**Conclusions:**

In septic shock patients, selepressin 2.5 ng/kg/minute was able to rapidly replace norepinephrine while maintaining adequate MAP, and it may improve fluid balance and shorten the time of mechanical ventilation.

**Trial registration:**

ClinicalTrials.gov, NCT01000649. Registered on September 30, 2009.

**Electronic supplementary material:**

The online version of this article (doi:10.1186/s13054-017-1798-7) contains supplementary material, which is available to authorized users.

## Background

Norepinephrine has traditionally been the vasopressor of choice in the treatment of septic shock, recommended as the first-line vasopressor in the Surviving Sepsis Guidelines [1]. However, vasopressin infusion has been used to replace norepinephrine to maintain adequate systemic arterial pressure (e.g., in patients refractory to norepinephrine) [2–4]. In a large, multicenter, randomized, double-blind, norepinephrine-controlled trial (the Vasopressin and Septic Shock Trial [VASST]), vasopressin decreased mortality compared with norepinephrine in patients with less severe septic shock, although the overall mortality was not different [5]. The researchers in the VAsopressin versus Noradrenaline as Initial therapy in Septic sHock (VANISH) randomized controlled trial of vasopressin vs. norepinephrine found no differences in rates of acute kidney injury, the primary endpoint [6]. Vasopressin infusion is currently recommended as a second-line vasopressor for septic shock in the Surviving Sepsis Guidelines [1] and is used clinically [7].

Selepressin, a novel, selective vasopressin V_1A_ receptor agonist, is a potent vasopressor, and it has also been shown to reduce fluid requirements and limit edema formation in animal septic shock models [8–11] and is now in clinical development for the treatment of septic shock. In a phase I first-in-human trial, selepressin infusion in 30 healthy subjects with infusion rates up to 3.0 ng/kg/minute for 6 h showed V_1A_-agonistic vasopressor properties, was safe and well tolerated, and showed no signs of vasopressin V_2_ activity (Ferring Pharmaceuticals A/S, unpublished data). In this first-in-patient pilot phase IIa randomized, placebo-controlled trial, the hypothesis was that selepressin maintains adequate arterial pressure in the absence of norepinephrine and shortens the duration of organ dysfunction in patients with early septic shock.

## Methods

### Study design

This was a multicenter, double-blind, randomized, placebo-controlled phase IIa trial investigating three ascending doses of selepressin in patients with early septic shock. Patients were recruited into the trial between 2009 and 2011 in Belgium, Denmark, and the United States in accordance with the Declaration of Helsinki and the principles of good clinical practice. The study protocol and informed consent documents were approved by the independent ethics committees or research ethics boards of all participating institutions, and written informed consent was obtained from all patients, their next of kin, or another surrogate decision maker as appropriate prior to enrollment. The study was approved by the competent regulatory authorities of each country participating in the trial. An independent data safety and monitoring committee evaluated the safety of the dose regimens prior to escalating to the next dose level.

At each dose level, patients were randomized to constant intravenous infusion of selepressin or placebo in a ratio of 2:1. Open-label norepinephrine was concomitantly administered to maintain the treatment target MAP of ≥65 mmHg. Study drug infusion continued as long as arterial pressure support was deemed necessary, but no longer than 7 days. Patients needing vasopressor support after 7 days were switched from study drug infusion to norepinephrine or another vasopressor. Assessments were performed during study drug treatment and up to 4 weeks after study drug initiation.

### Study population

Patients with hypotension in early septic shock, defined as hypotension not responding to infusion of fluid and requiring at least 0.1 μg/kg/minute norepinephrine for at least 2 h, with a proven or suspected site of infection and at least one sign of tissue hypoperfusion (oliguria, decreased Glasgow Coma Scale score, decreased ratio of partial pressure arterial oxygen to fraction of inspired oxygen, or increased arterial blood lactate) could be included. To be eligible, patients had to be shifted to the open-label norepinephrine and randomized to a constant intravenous infusion of selepressin or placebo within 24 h of meeting the inclusion criteria. Briefly, exclusion criteria (*see* Additional file 1: Table S1 for details) were acute coronary syndrome; hypovolemia suspected on clinical grounds; cardiac failure; pregnancy or breastfeeding; hypotension other than septic shock; use of vasopressin or terlipressin during the current hospital admission; acute mesenteric ischemia; episode of septic shock within 1 month; death anticipated within 24 h; chronic heart disease, including heart failure and second- and third-degree atrioventricular block without pacemaker; hyponatremia; brain injury; burn; peripheral vascular disease; previously randomized in this trial; intake of an investigational drug within the last 3 months; participation in another clinical trial; and considered unsuitable to participate in the trial for any other reason.

### Sample size and randomization

The study design comprised four treatment cohorts at three ascending infusion rate levels, the first three cohorts with ten receiving active treatment and five receiving placebo. The last cohort comprised active and placebo to finally reach 20 patients at the maximum tolerated infusion level and 20 patients receiving placebo. The randomization process was a computer-generated random listing of the treatment allocations, stratified by center and in variable permuted blocks of 2, 4, or 6.

### Infusion of study drug and norepinephrine

The investigated starting and maximal infusion rates of selepressin were 1.25 ng/kg/minute, 2.5 ng/kg/minute, and 3.75 ng/kg/minute, with the patient’s body weight being measured or estimated. Selepressin or placebo was infused via a central venous catheter at the constant initial rate in addition to open-label norepinephrine targeting a mean arterial pressure (MAP) of ≥65 mmHg. When patients were hemodynamically stable, open-label norepinephrine was tapered while maintaining target MAP with study drug. When the MAP was stable for 4 h without norepinephrine, study drug was weaned stepwise according to the protocol (Additional file 1: Table S2). If weaning resulted in hemodynamic instability, the study drug was reinstituted, and, if needed, open-label norepinephrine was restarted. Open-label norepinephrine was adjusted to maintain the MAP ≥65 mmHg if blinded study drug was insufficient. Patients received study drug until shock resolution (i.e., no vasopressor support) or a maximum of 7 days unless discontinued for safety reasons (Additional file 1: Table S3).

Open-label norepinephrine was supplied in the United States and Canada as norepinephrine bitartrate 1 mg/ml (Levophed, norepinephrine base; Hospira, Lake Forest, IL, USA) diluted in 5% dextrose and in Europe as Norepinephrine 1:1000 (norepinephrine tartrate; (Cardinal Health Ltd., Basingstoke, UK) 2 mg/ml (1 mg/ml norepinephrine base) diluted in 5% glucose. A tight protocol and accurate pumps (Braun Perfusor® Space; B. Braun Melsungen AG, Melsungen, Germany) were used to calculate open-label norepinephrine delivered.

### Study outcomes

The co-primary endpoints were stabilization of MAP as determined by proportion of patients maintaining a MAP >60 mmHg without open-label norepinephrine at 12, 24, 48, and 96 h, and day 7; infusion rates and cumulative dose of open-label norepinephrine; and proportion of patients maintaining a MAP >60 mmHg at 12, 24, 48, and 96 h, and day 7, regardless of open-label norepinephrine administration. The limit for maintained MAP was defined as 60 mmHg to prevent small variations around the clinical treatment target of 65 mmHg having disproportional impact on the overall evaluation.

Secondary endpoints included pharmacodynamics, pharmacokinetics, safety (vital signs, central venous pressure, central venous oxygen saturation, electrocardiographic and cardiac function, respiratory function, clinical chemistry, hematology, hemostasis, and urinalysis), organ dysfunction, an indirect measure of vascular leakage (i.e., fluid balance), and morbidity. Treatment-emergent adverse events (occurrence from start of study drug to 48 h after infusion was stopped) were collated and evaluated. Morbidity and organ dysfunction were evaluated by time course of Sequential Organ Failure Assessment (SOFA) scores; days alive and free of organ dysfunction (using SOFA); proportion of patients off all vasopressors; days alive and out of intensive care unit (ICU); days alive and free of vasopressors; corticosteroids for sepsis treatment, dialysis, or mechanical ventilation at days 7, 14, and 28; ICU and hospital (up to day 28) lengths of stay; and plasma C-reactive protein (CRP), tumor necrosis factor (TNF)-α, interleukin (IL)-6, IL-10, and IL-1ra levels.

### Pharmacokinetics

Because the study drug infusion was administered according to need, the pharmacokinetic parameters steady-state concentration, total systemic clearance, distribution volume at steady state, and half-life were calculated by modeling using a two-compartment population pharmacokinetic model with random subject effects on clearance and distribution volume using WinNonlin®Pro (Pharsight Corp., Cary, NC, USA). Actual blood sampling time points were used for the calculations.

### Statistical analysis

All statistical analyses were performed using SAS version 9.2 for Windows software (SAS Institute Inc., Cary, NC, USA). The first two co-primary endpoints were compared using a logistic regression model. The rates and cumulative amounts of open-label norepinephrine administered were compared between treatment groups by using a repeated measures analysis of covariance (ANCOVA) model with treatment, time, and treatment by time interaction as factors; baseline rate of norepinephrine as a covariate; and subject as the experimental unit. The analyses were done for both the full analysis set and the per-protocol analysis set. For both analysis sets, the analyses were presented for the whole analysis set and those alive (and not discontinued) at the time of the measurement. The selepressin groups were compared individually with the placebo group in an analysis of variance model.

Changes in vascular leakage endpoints (i.e., fluid balance), as well as changes from baseline in cytokines and SOFA scores, were compared between treatment groups using the same ANCOVA model as for norepinephrine. Patients were categorized as free of organ dysfunction if all six individual SOFA scores were 0. Percentage days alive and free of organ dysfunction/ICU/hospital (i.e., number of days/7 observation days × 100) were analyzed between treatment groups using nonparametric Wilcoxon tests. The treatment differences between the selepressin groups and placebo were estimated using the Hodges-Lehmann estimator for independent samples. The proportions of patients alive were analyzed between treatment groups on days 7, 14, and 28 using a logistic regression model. Confidence intervals (Clopper-Pearson) were calculated for mortality rates and for the ORs of mortality rates.

## Results

### Study population

**Fig. 1:**
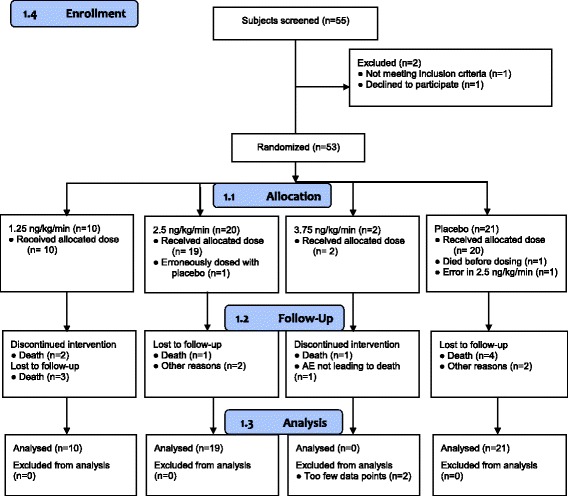
Consolidated Standards of Reporting Trials (CONSORT) diagram of patient flow through the study

Fifty-three patients were randomized, and 52 subjects were dosed; 10 subjects received 1.25 ng/kg/minute, 19 subjects received 2.5 ng/kg/minute, 2 subjects received 3.75 ng/kg/minute, and 21 subjects received placebo. All randomized patients were included in the intention-to-treat dataset. Two patients were infused at 3.75 ng/kg/minute, one of whom had the study drug infusion discontinued for possible safety reasons, with subsequent discontinuation of this dose group. Owing to the small sample size and short duration of infusion in this group, efficacy analyses were not possible. A Consolidated Standards of Reporting Trials diagram of the study is shown in Fig. 1.

There were essentially no differences between selepressin- and placebo-treated patients in baseline characteristics, apart from a lower baseline norepinephrine dose in the 1.25 ng/kg/minute than in the 2.5 ng/kg/minute and placebo groups (Table 1). The most common underlying organ dysfunctions were gastrointestinal, metabolic and nutritional, respiratory, and renal (Table 1). The primary infection was predominantly abdominal (40%) or pulmonary (31%).

**Table 1:** Demographic and baseline characteristics of patients with septic shock

	Selepressin 1.25 ng/kg/minute (*n* = 10)	Selepressin 2.5 ng/kg/minute (*n* = 19)	Selepressin 3.75 ng/kg/minute (*n* = 2)	Placebo (*n* = 21)
Demographics				
Sex				
Female/male, *n* (%)	7 (70%)/3 (30%)	9 (47%)/10 (53%)	1 (50%)/1 (50%)	6 (29%)/15 (71%)
Age, years, mean (SD; median)	59.3 (19.6–62.5)	57.1 (15.4–59)	69 (12.7–69)	63.2 (18–66)
Weight, kg, mean (SD; median)	64.8 (14.3–63)	87.6 (28.6–85)	77.5 (17.7–78)	75.1 (15.3–75)
Total SOFA score, mean (SD; median)	9.3 (2.2–9.5)	11.2 (3.7–11)	12 (0: 12)	10.4 (3.5–11)
Lactate, mEq/L, mean (SD)	2.5 (1.5)	3.0 (2.9)	7.5 (8.1)	2.5 (1.4)
Mean arterial pressure, mmHg, mean (SD; median)	66 (13–63)	74 (9–70)	69 (15–69)	74 (13–69)
Heart rate, beats/minute, mean (SD; median)	90 (17–94)	97 (20–91)	110 (6–110)	90 (20–93)
Norepinephrine, μg/kg/minute, mean (SD)	0.18 (0.09)	0.28 (0.26)	0.39 (0.20)	0.34 (0.35)
PaO_2_/FiO_2_, mean (SD; median)	231 (108–200)	257 (133–233)	221 (24–221)	246 (129–198)
Primary infection type				
Bacterial, *n* (%)	7 (70%)	14 (74%)	2 (100%)	15 (71%)
Unknown, *n* (%)	3 (30%)	5 (26%)		5 (24%)
Other, *n* (%)				1 (5%)
Primary infection location				
Urinary tract, *n* (%)	1 (10%)	2 (11%)		1 (5%)
Lung, *n* (%)	1 (10%)	6 (32%)	1 (50%)	8 (38%)
Abdomen, *n* (%)	4 (40%)	8 (42%)		9 (43%)
Unknown, *n* (%)	2 (20%)			1 (5%)
Other, *n* (%)	2 (20%)	3 (16%)	1 (50%)	2 (10%)
Main concomitant diseases				
Gastrointestinal, *n* (%)	8 (80%)	14 (74%)	1 (50%)	20 (95%)
Metabolic, *n* (%)	6 60%)	16 (84%)	2 100%)	18 (86%)
Respiratory, *n* (%)	7 (70%)	12 (63%)	2 100%)	14 (67%)
Renal, *n* (%)	5 (50%)	11 (58%)	1 (50%)	16 (76%)
Subjects on mechanical ventilation	4 (40%)	11 (58%)	0	15 (71%)

*PaO*_*2*_ */FiO*_*2*_ Ratio of partial pressure arterial oxygen and fraction of inspired oxygen, *SOFA* Sequential Organ Failure Assessment

MAPs during the 7-day assessment period were similar between groups at approximately 70 mmHg initially and increasing over 2 days to approximately 80–85 mmHg (Additional file 1: Figure S1).

### Effect of selepressin on norepinephrine requirement and duration of septic shock

**Fig. 2:**
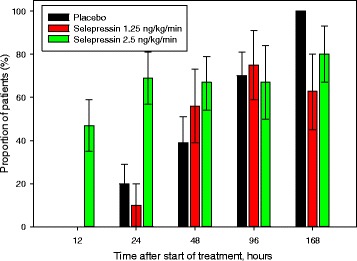
Proportion of patients maintaining a mean arterial pressure >60 mmHg without any open-label norepinephrine support at the indicated time points. The difference between 2.5 ng/kg/minute and placebo was statistically significant (p < 0.01) at 24 h. Results are means, with bars indicating SD

The mean total selepressin infused doses were 6.1 and 8.7 μg/kg infused over 3.6 and 3.2 days in the 1.25 and 2.5 ng/kg/minute dose groups, respectively. The swift onset of action of selepressin was illustrated by the high proportion of patients receiving 2.5 ng/kg/minute selepressin who early on maintained MAP >60 mmHg without norepinephrine (about 50% at 12 h and 70% at 24 h) (Fig. 2). In contrast, in the placebo and 1.25 ng/kg/minute selepressin groups, no patient was free of norepinephrine at 12 h and ≤20% were free of norepinephrine at 24 h (*p* < 0.01). However, over time, the differences decreased as more patients recovered also in the latter groups.

**Fig. 3:**
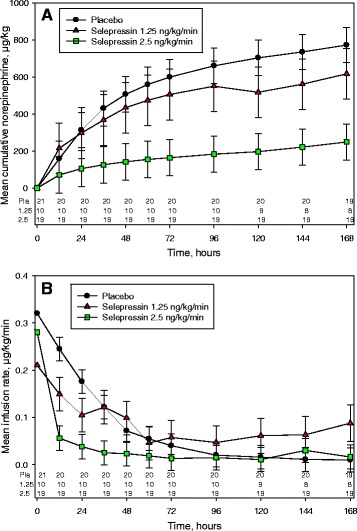
Mean cumulative amount (**a**) and infusion rate (**b**) of open-label norepinephrine over time in septic shock patients. Selepressin and placebo were infused at a constant rate as indicated, whereas norepinephrine was weaned as fast as possible while still keeping the target treatment mean arterial pressure ≥65 mmHg. Numbers at the bottom of the figure indicate number of patients at each time point. Bars indicate SD. Pl Placebo, 1.25 1.25 ng/kg/minute, 2.5 2.5 ng/kg/minute

The 7-day baseline adjusted mean cumulative doses of open-label norepinephrine were 761, 659, and 249 μg/kg for the placebo, 1.25 ng/kg/minute, and 2.5 ng/kg/minute groups, respectively (*p* < 0.001 for 2.5 ng/kg/minute vs. placebo groups) (Fig. 3a). Furthermore, the norepinephrine mean infusion rate was initially reduced more rapidly in the selepressin 2.5 ng/kg/minute group than in the placebo group; at 24 h, the infusion rates were 0.04 vs. 0.18 μg/kg/minute (*p* < 0.005) (Fig. 3b).

As expected, there were no differences between treatment groups in the proportions of patients who maintained MAP >60 mmHg regardless of norepinephrine, meaning that the patients were generally treated equally (Additional file 1: Table S4). Selepressin at 2.5 ng/kg/minute appeared to result in a faster recovery from shock (off vasopressors, including selepressin) than 1.25 ng/kg/minute and placebo, but without statistical significance (58% vs. 29%, *p* = 0.11, at 48 h) (Additional file 1: Figures S2 and S3). There was no significant difference between groups in 28-day mortality rates (placebo 4 [21%] of 19, selepressin 1.25 ng/kg/minute 5 [50%] of 10, selepressin 2.5 ng/kg/minute 1 [5%] of 19).

### Effect of selepressin on mechanical ventilation, fluid balance, and other morbidity

The proportion of days alive and free of ventilation was greater in the selepressin 2.5 ng/kg/minute group than in the placebo group (54% vs. 23%, *p* < 0.02) over the 7-day treatment period. There was no difference between the 1.25 ng/kg/minute (31%) and placebo groups (Additional file 1: Figure S4).

**Fig. 4:**
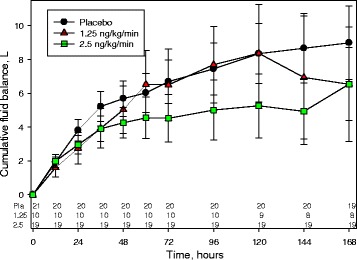
Mean cumulative net fluid balance over 7 days from start of selepressin/placebo treatment. Selepressin and placebo were infused at a constant rate as indicated. Numbers at the bottom of the figure indicate number of patients at each time point. Bars indicate SD. Pl Placebo, 1.25 1.25 ng/kg/minute, 2.5 2.5 ng/kg/minute

Selepressin at 2.5 ng/kg/minute decreased the cumulative net fluid balance over the treatment period compared with the 1.25 ng/kg/minute and placebo groups (from about 9 L to 6.5 L, *p* = 0.1) (Fig. 4), and compared with placebo, the difference was significant (*p* < 0.05) from day 5 (94 h) onward. The differences in fluid balance appeared to be due to fluid input rather than to urine output because there were no differences between groups in urine output (Additional file 1: Figure S5).

There were no significant differences between groups in length of stay in the ICU or hospital up to 28 days; in plasma CRP, TNF-α, IL-6, IL-10, or IL-1ra levels; or in any other secondary morbidity endpoint.

### Pharmacokinetics

The mean steady-state concentrations of selepressin (0.50 and 0.99 ng/ml) were proportional to the initial infusion rates of 1.25 and 2.5 μg/kg/minute, with a time to steady-state concentration of approximately 7 h. The modeled mean total systemic clearance values were 10.0 and 13.1 L/h, respectively, increasing with body weight and being higher in men than in women. The terminal half-life was approximately 2.5 h in both dose groups, with an initial distribution/elimination phase half-life of approximately 10 minutes. The distribution volume at steady state was 18–31 L, indicating extravascular distribution (Additional file 1: Table S5).

### Safety

Selepressin was well tolerated, with no difference between selepressin dose groups and placebo in terms of treatment-emergent adverse events. The high-dose group was stopped after two patients because of potential adverse events in the second patient. The most frequent adverse events were atrial fibrillation, bradycardia, and hypertension (six subjects on seven occasions), equally distributed across treatment groups. The severe treatment-emergent adverse events were generally single observations attributable to the underlying disease (Additional file 1: Table S6). Nine treatment-emergent adverse events (in eight subjects) were regarded by the investigator to be related to treatment; one, four, and four treatment-emergent adverse events were regarded as mild, moderate, and severe, respectively (Table 2). Four of the adverse drug reactions in three patients were judged as serious: myocarditis and peripheral ischemia (one patient in the 2.5 ng/kg/minute group), myocardial ischemia (3.75 ng/kg/minute group), and atrial fibrillation (placebo group). There were no deaths related to ischemic event(s) attributable to selepressin.

**Table 2:** Treatment-emergent adverse drug reactions that were possibly or probably related to treatment

	Selepressin 1.25 ng/kg/minute (*n* = 10)	Selepressin 2.5 ng/kg/minute (*n* = 19)	Selepressin 3.75 ng/kg/minute (*n* = 2)	Placebo (*n* = 21)
	No. of subjects (%), events	No. of subjects (%), events	No. of subjects (%), events	No. of subjects (%), events
Cardiac disorder				
Atrial fibrillation				1 (5), 1
Cyanosis		1 (5), 1		
Myocardial ischemia			1 (50), 1	
Myocarditis		1 (5), 1		
Metabolism and nutritional disorders				
Hyperlactatemia				1 (5), 1
Skin and subcutaneous tissue disorders				
Macular rash				1 (5), 1
Vascular disorders				
Hypertension		2 (11), 2		
Peripheral ischemia		1 (5), 1		

There were no regional or global signs of hypoperfusion, as suggested by similar decreases of lactate levels (Additional file 1: Figure S5) and serum creatinine (Additional file 1: Figure S6), with no significant differences between study groups during the treatment period.

## Discussion

In this phase IIa trial in patients in septic shock, the selective V_1A_ agonist selepressin was shown to be an effective vasopressor because infusion of 2.5 ng/kg/minute maintained an adequate MAP, increased the proportion of patients weaned off norepinephrine during the first 24 h, and decreased the mean cumulative dose of norepinephrine, thus demonstrating rapid onset and sustained activity. Moreover, 2.5 ng/kg/minute selepressin could possibly mitigate lung dysfunction because it was associated with a higher proportion of days alive and free of ventilation over 7 days than placebo. It is well known that long duration of mechanical ventilation increases the risk of nosocomial pneumonia, neuromuscular weakness, and death. Also, although not statistically significant, twice as many patients were out of shock within 48 h with selepressin 2.5 ng/kg/minute compared with placebo, suggesting potential clinical benefit. Thus, selepressin appears to shorten duration of shock and the time with mechanical ventilation and may be expected to potentially improve the overall treatment outcome.

Increased vascular leakage due to increased endothelial permeability in septic shock results in edema and organ dysfunction, and increased positive net fluid balance is directly associated with long duration of mechanical ventilation [12] and increased mortality in sepsis and septic shock [13–15]. An indirect marker of vascular leakage—cumulative fluid balance over 7 days—was lower with selepressin 2.5 ng/kg/minute than with placebo, a result that is consistent with animal models of septic shock [8–11]. In a prospective randomized study in an ovine *Pseudomonas aeruginosa* pneumonia model of septic shock of selepressin vs. Vasopressin, researchers found that selepressin blocked vascular leak better than vasopressin did and that the decreased vascular leakage was reversed by adding the V_2_ agonist ddAVP to selepressin [9]. Taken together, these results suggest that selective vasopressin V_1A_ agonism may protect against increased vascular leakage in septic shock.

The V_1A_-selective activity of selepressin may also have other advantages compared with nonselective vasopressin agonists in septic shock. Terlipressin is more of a V_1A_ receptor agonist than vasopressin, but it is also a V_1B_ and V_2_ receptor agonist, whereas selepressin is a highly selective V_1A_ receptor agonist. In contrast to vasopressin and terlipressin, selepressin does not activate the vasopressin V_2_ receptors that are mediating antidiuretic effects [16], release of von Willebrand factor [16, 17], and vasodilation by stimulation of nitric oxide production [18, 19], all of which might be harmful during septic shock due to exacerbation of oliguria, procoagulation, and vasodilation.

Selepressin at 1.25 ng/kg/minute had only very limited pressor effects, suggesting that 2.5 ng/kg/minute was required for an effective discontinuation of norepinephrine infusion. There was no difference between selepressin dose groups and placebo in terms of treatment-emergent adverse events. However, the highest-dose group was stopped after two patients because of adverse events in one of the patients that were regarded to be possibly related to selepressin. Because selepressin 3.75 ng/kg/minute was discontinued following only two patients with short infusion times, it was not possible to assess the safety and efficacy of this dose. However, 3.75 ng/kg/minute and higher infusion rates did not raise any safety concerns in a separate uncontrolled, multicenter, open-label trial in which all 30 patients received selepressin at infusion rates of 3.75 ng/kg/minute and higher (Ferring Pharmaceuticals A/S, unpublished data). Further assessment of the safety and efficacy of selepressin (at infusion rates of 1.7–7.5 ng/kg/minute) vs. placebo is currently ongoing in the phase IIb/III Selepressin Evaluation Programme for Sepsis-Induced Shock - Adaptive Clinical Trial (SEPSIS-ACT; clinicaltrials.gov/ct2/show/NCT02508649?term = selepressin&rank = 1).

Selepressin clearance was about 30% lower in septic shock patients than in healthy subjects. Because peptides similar in size are eliminated predominantly by peptidase degradation and excretion in the kidney, the decreased clearance in septic shock may be explained by reduced renal function. Accordingly, the terminal half-life of selepressin was longer in septic shock patients (2.5 h) than in healthy subjects (1.5 h) (Ferring Pharmaceuticals, unpublished data). The longer half-life of selepressin than of norepinephrine would potentially limit rapid adjustment of the selepressin-induced vasopressor support, especially after steady state has been established. However, the selepressin initial distribution/elimination phase half-life was short (10 minutes), enabling rapid selepressin dose adjustment during the early phase. Weaning of vasopressor support is generally not rapid; there were no indications that the terminal half-life caused undue vasopressor-related events. In comparison with vasopressin (short half-life) and terlipressin (long half-life) [20, 21], selepressin’s half-life is intermediate [22, 23].

Infusion of selepressin at 2.5 ng/kg/minute appeared safe and was well tolerated, as shown by the similar frequency of treatment-emergent adverse events in the selepressin at 2.5 ng/kg/minute and placebo groups. The treatment-emergent adverse events related to selepressin could generally be attributed to vasoconstriction and were similar in the selepressin and placebo groups, as were occurrence of severe and serious adverse events. There were no differences between groups in indirect markers of regional or global signs of hypoperfusion (lactate and serum creatinine levels). The pooled mortality of the selepressin groups was 20%, very similar to the 21% placebo group mortality rate and consistent with mortality rates in recent trials in septic shock [24]. The small sample size and the wide variability in clinical presentations and outcomes of septic shock prevented any conclusions on mortality, but none of the deaths was regarded as related to selepressin.

This was the first trial in patients, and the overall goal of the trial was to determine whether and to what degree selepressin could decrease the dose of norepinephrine in septic patients requiring vasopressor support. Reducing the dose of norepinephrine—so-called “de-catecholaminization” [25]—could in itself be advantageous because it could decrease the adverse effects of norepinephrine, including excessive prearteriolar vasoconstriction (compared with vasopressin [26] and possibly selepressin) and tachyarrhythmias; it could have beneficial effects on fluid balance and vascular leak [8, 9]; and it could possibly have more adverse effects on immunity [27] than selepressin’s selective V_1A_ agonism.

It remains to be determined whether rapid and full substitution of norepinephrine with selepressin is superior to cotreatment with norepinephrine and selepressin. That will be assessed in the ongoing phase IIb/III SEPSIS-ACT trial. The combination of vasopressin and norepinephrine possesses synergistic effects, and the two different modes of action together may be superior to aiming to reach target MAP fully with either strategy alone. However, the potential additional benefits of selepressin compared with vasopressin, such as reduction of capillary leakage and lack of V_2_-mediated antidiuresis and procoagulation activity, may justify earlier use and fuller substitution of norepinephrine with selepressin.

### Strengths and weaknesses of this trial

Strengths of this trial include the randomized, concealed, placebo-controlled design; generalizability (multicenter in Europe and North America); precision of the co-primary endpoints; the inclusion of patients with relatively early septic shock; the novel selective V_1A_ agonist selepressin; and administration of a tight protocol in critically ill septic shock patients. Limitations of this trial include the small sample size due to the early stage in selepressin’s clinical development in a limited number of centers (that may limit the overall impact), but they were suitable for initial assessment of selepressin in human septic shock. The fluid input was not controlled, and we do not have information about the different solutions used (crystalloids, colloids, blood products), which might, at least in part, have influenced the amount of infused fluids, a potential limitation in interpreting the fluid balance. Although the addition of selepressin allowed sparing of norepinephrine and some would argue that one is simply substituting one vasopressor (selepressin) for another (norepinephrine), the beneficial effects on ventilation and fluid balance suggest additional nonvasopressor benefits of selepressin vs. norepinephrine. The pharmacodynamic effects of selepressin should be interpreted with caution owing to the small sample size of this phase IIa trial. We did not measure cardiac output, so we cannot comment on selepressin vs. norepinephrine effects on this parameter. Of note, vasopressin and norepinephrine had similar effects—no decrease in cardiac output in VASST [28]. Interaction of vasopressin and corticosteroids has been reported elsewhere, but owing to the small sample size, it was not possible to assess the potential interaction of corticosteroids and selepressin in this trial.

## Conclusions

The novel selective vasopressin V_1A_ receptor agonist selepressin at an infusion rate of 2.5 ng/kg/minute rapidly replaced norepinephrine while maintaining target MAP and may have improved fluid balance and shortened the time of mechanical ventilation. Further studies of selepressin’s mechanism of action and additional larger randomized controlled trials to investigate its efficacy are needed and ongoing to assess its ability to improve the treatment outcome of patients in septic shock.

## Additional file

Additional file 1: Table S1. Exclusion criteria. **Table S2.** Study drug discontinuation criteria. **Table S3.** Study drug weaning protocol. **Table S4.** Proportion of patients maintaining MAP >60 mmHg. **Table S5.** Pharmacokinetic parameters of selepressin in septic shock patients. **Table S6.** Summary of treatment emergent severe adverse events by system organ class and preferred term. **Figure S1.** Mean arterial pressure over time. **Figure S2.** Proportion of patients out of shock during the first 72 h of treatment with selepressin or placebo. **Figure S3.** Kaplan-Mayer estimation of time to septic shock resolution. **Figure S4.** Proportion of days alive and off ventilator during the 7-day period from start of selepressin/placebo treatment. **Figure S5.** Mean cumulative urine output over time in septic shock patients. **Figure S6.** Mean lactate over time in septic shock patients. **Figure S7.** Mean creatinine over time in septic shock patients. (DOCX 138 kb)
