# Supplementary material for: Selepressin, a novel selective vasopressin V1A agonist, is an effective substitute for norepinephrine in a phase IIa randomized, placebo-controlled trial in septic shock patients
Source: Crit Care. 2017 Aug 15;21:213. doi: 10.1186/s13054-017-1798-7 (PMC5557574; doi:10.1186/s13054-017-1798-7)
Supplement: Additional file 1: Table S1. — Exclusion criteria. Table S2. Study drug discontinuation criteria. Table S3. Study drug weaning protocol. Table S4. Proportion of patients maintaining MAP >60 mmHg. Table S5. Pharmacokinetic parameters of selepressin in septic shock patients. Table S6. Summary of treatment emergent severe adverse events by system organ class and preferred term. Figure S1. Mean arterial pressure over time. Figure S2. Proportion of patients out of shock during the first 72 h of treatment with selepressin or placebo. Figure S3. Kaplan-Mayer estimation of time to septic shock resolution. Figure S4. Proportion of days alive and off ventilator during the 7-day period from start of selepressin/placebo treatment. Figure S5. Mean cumulative urine output over time in septic shock patients. Figure S6. Mean lactate over time in septic shock patients. Figure S7. Mean creatinine over time in septic shock patients. (DOCX 138 kb) [file 13054_2017_1798_MOESM1_ESM.docx]

**SELEPRESSIN, A NOVEL SELECTIVE VASOPRESSIN V_1A_ AGONIST, EFFECTIVELY SUBSTITUTES NOREPINEPHRINE IN A PHASE 2A RANDOMIZED PLACEBO-C0NTROLLED TRIAL IN SEPTIC SHOCK PATIENTS**

**JAMES A. RUSSELL^1^ MD, PhD, ^,^ JEAN-LOUIS VINCENT^2^, MD, PhD,**

**ANNE LOUISE KJØLBYE^3^, MSC, PhD, MBA, HÅKAN OLSSON^3^, PhD,**

**ALLAN BLEMINGS^3^, MSc, HERBERT SPAPEN^4^, MD, PhD, PEDER CARL^5^, MD, PhD, PIERRE-FRANCOIS LATERRE^6^, MD, PhD LARS GRUNDEMAR^3^, MD, PhD**

**SUPPLEMENTAL DIGITAL CONTENT**

**Additional file 1: Table S1.**

**Inclusion and Exclusion Criteria**

**Inclusion Criteria**

All of the following inclusion criteria must be met before the subject can enter into the trial:

1. Signed informed consent form by the patient or a legal representative according to local regulations
2. Man or woman 18 years of age or older
3. Proven or suspected infection
4. Hypotension (systolic blood pressure less than 90 mmHg or decrease in systolic blood pressure by at least 40 mmHg for more than one hour) that has not responded to fluid (20 ml/kg of crystalloid or 10 ml/kg of colloid), requiring norepinephrine at a dose of at least 0.1 μg/kg/min for at least 2 hours
5. Signs of tissue hypoperfusion (at least one of the following criteria): Oliguria (< 0.5 ml/kg/hr for 1 hour), decreased Glasgow Coma Score (< 13), decreased PaO2/FiO2 ratio (< 300), increased arterial blood lactate (> 4 mmol/L). Patients with low Glasgow Coma Score that can be ascribed to sedation and patients with decreased PaO2/FiO2 ratio secondary to pneumonia should have one additional sign of tissue hypoperfusion
6. Willing to use an adequate barrier method or hormonal method of contraception, if not abstinent, from informed consent to one week after the end of infusion of study medication

**Exclusion Criteria**

The exclusion criteria are as follows:

1. Present or a history (within the last 5 years) of acute coronary syndrome (myocardial infarction or unstable angina). Patients who have been asymptomatic for 6 months after coronary revascularisation were eligible. Known or suspected coronary ischemia.
2. Hypovolemia suspected on clinical grounds, e.g. cold extremities with delayed capillary filling, low cardiac filling pressure, marked systolic or pulse pressure variation or positive leg raising test
3. Known or suspected cardiac failure, e.g. CI < 3 l/min/m^2^, left ventricular ejection fraction (LVEF) ≤40%, bilateral pulmonary oedema/congestion and cardiomegaly, ScvO_2_ (superior vena cava) <64%, known or suspected coronary ischemia, cold extremities with delayed capillary filling, distended neck veins.
4. Pregnancy or breastfeeding
5. Any cause of hypotension other than early septic shock
6. Use of vasopressin or terlipressin for blood pressure support during the current hospital admission
7. Proven or suspected acute mesenteric ischemia, as judged by the investigator
8. Known episode of septic shock within 1 month prior to randomisation
9. Death anticipated within 24 hours
10. Underlying chronic heart disease, including heart failure NYHA class III or IV
11. Known past or current 2^nd^ and 3^rd^ degree AV-block without a well-functioning pacemaker
12. Hyponatremia (serum/plasma sodium <130 mmol/L)
13. Traumatic brain injury (Glasgow Coma Score <8 prior to onset of sepsis)
14. Present hospitalisation with burn injury
15. Symptomatic peripheral vascular disease including Raynaud’s syndrome
16. Previously randomised in this trial
17. Intake of an investigational drug within the last 3 months (or longer if judged by the Investigator to possibly influence the outcome of the current study)
18. Known participation in another clinical trial
19. Considered by the investigator to be unsuitable to participate in the trial for any other reason.”

###

**Additional file 1: Table S2. Study drug weaning protocol**

When patients are hemodynamically stable, i.e. MAP is stable at the target, open label norepinephrine will be tapered off in accordance with local clinical practice while infusion of study drug continues. If MAP exceeds 70 mmHg for an hour after open label norepinephrine is stopped, the infusion rate of study drug is reduced every 4 hours (or hourly if MAP >80 mmHg) stepwise by 20% to keep the target of 65 mmHg. The infusion rate of study drug can only be reduced when the open label NE is no longer required and the infusion has been stopped. As long as the MAP remains at target the infusion rate of study drug continues unchanged.

### Additional file 1: Table S3.

### Study Drug Discontinuation Criteria

Study drug infusion was permanently discontinued if any of the following occurred:

- Signs of coronary ischemia as shown by 12-lead ECG (S-T segment and/or Q-wave changes) and troponin T or troponin I elevation, or troponin T or I elevation in combination with other clinical or laboratory findings indicative of myocardial ischemia
- Serious or life-threatening (haemodynamically unstable) cardiac arrhythmias
- Development of 2^nd^ or 3^rd^ degree AV-block without a well-functioning pacemaker
- Signs of acute mesenteric or hepatic ischemia.
- Clinically relevant digital ischemia.
- Hyponatremia (serum sodium <125 mmol/L)
- If the investigator considers this to be in the best interest of the patient, e.g. if a non-allowed treatment was required.

If any of these adverse events occurred, the infusion of study medication was permanently stopped. Any occurrence of these events was to be reported as a serious adverse event (SAE) and treated at the discretion of the investigator. The Sponsor, and the International Signatory Investigator in agreement with the Sponsor, reserved the right to discontinue the trial at any time for safety reasons or other reasons jeopardising the justification of the trial. The subjects had the right to withdraw from the trial at any time for any reason, without the need to justify their decision.

**Additional file 1: Table S4.** **Proportion^1^ of patients maintaining target MAP (>60 mmHg) with or without vasopressors.**

| **Time after start of infusion** |  | **Selepressin 1.25 ng/kg.min** | **Selepressin 2.5 ng/kg.min** | **Placebo** |
| --- | --- | --- | --- | --- |
| **Day 1 12 hours** | Proportion, % 95% confidence interval^2^ | 100% 63; 100 | 100% 81; 100 | 95% 94; 100 |
| **Day 1 24 hours** | Proportion, % 95% confidence interval^2^ | 88% 48; 100 | 100% 78; 100 | 100% 83; 100 |
| **Day 2 48 hours** | Proportion, % 95% confidence interval^2^ | 100% 66; 100 | 100% 77; 100 | 100% 82; 100 |
| **Day 4 96 hours** | Proportion, % 95% confidence interval^2^ | 100% 63; 100 | 100% 63; 100 | 100% 81; 100 |
| **Day 7 168 hours** | Proportion, % 95% confidence interval^2^ | 83% 36; 100 | 100% 66; 100 | 100% 78; 100 |

^1^Proportion is based on those alive at the time of measurement, i.e. those discontinued are not considered part of the denominator

^2^Clopper-Pearson confidence interval

**Additional file 1: Table S5. Pharmacokinetic parameters of selepressin in septic shock patients**

| **PK parameter** | **1.25 ng/kg.min N=10** | **2.5 ng/kg.min N=17** |
| --- | --- | --- |
| Steady state concentration, ng/mL | 0.50 (0.13) | 0.99 (0.32) |
| Time to steady state, h | 7.6 (2.8) | 7.0 (2.3) |
| Clearance, L/h | 10.0 (2.8) | 13.1 (2.9) |
| Steady state volume, L | 25.5 (10.6) | 31.2 (14.7) |
| Initial elimination half-life, h | 0.18 (0.09) | 0.17 (0.06) |
| Terminal elimination half-life, h | 2.5 (1.1) | 2.7 (1.2) |

N=number of subjects included in calculations

Data are generated by modelling the concentration data available from all subjects. Mean (SD)

Additional file 1: Table S6. Summary of treatment emergent severe adverse events by system organ class and preferred term.

|  | **1.25 ng/kg.min**  **N=10** | **2.5 ng/kg.min**  **N=19** | **3.75 ng/kg.min**  **N=2** | **Placebo**  **N=21** |
| --- | --- | --- | --- | --- |
|  | N (%) E | N (%) E | N (%) E | N (%) E |
| **Any severe TEAE** | **3 (30) 8** | **4 (21) 6** | **2 (100) 2** | **8 (38) 16** |
|  | | | | |
| **Blood and lymphatic system disorders** | | | | |
| Disseminated intravascular coagulation | 1 (10) 1 |  |  |  |
| **Cardiac disorder** | | | | |
| Atrial fibrillation |  |  |  | 1 (5) 1 |
| Bradycardia |  |  |  | 1 (5) 1 |
| Cardio-respiratory arrest |  |  | 1 (50) 1 | 1 (5) 1 |
| Myocardial ischemia |  |  | 1 (50) 1 |  |
| Myocarditis |  | 1 (5) 1 |  |  |
| **Gastrointestinal disorders** | | | | |
| Ascites | 1 (10) 1 |  |  |  |
| Diarrhea |  |  |  | 1 (5) 1 |
| Gastrointestinal ischemia |  |  |  | 1 (5) 1 |
| **Hepatobiliary disorders** | | | | |
| Hepatic failure | 1 (10) 1 |  |  |  |
| **Infections and infestations** | | | | |
| Hematoma infection |  |  |  | 1 (5) 1 |
| **Injury, poisoning, and procedural complications** | | | | |
| Mechanical ventilation complication | 1 (10) 1 |  |  |  |
| **Investigations** | | | | |
| Oxygen saturation decreased |  |  |  | 1 (5) 1 |
| Troponin increased |  |  |  | 1 (5) 1 |
| **Metabolism and nutritional disorders** | | | | |
| Diabetes mellitus |  |  |  | 1 (5) 1 |
| Hyperkalemia | 1 (10) 1 |  |  | 1 (5) 1 |
| Metabolic acidosis |  |  |  | 1 (5) 1 |
| **Nervous system disorders** | | | | |
| Cerebral hemorrhage | 1 (10) 1 |  |  |  |
| **Psychiatric disorders** | | | | |
| Anxiety |  |  |  | 1 (5) 1 |
| **Renal and urinary disorders** | | | | |
| Oliguria | 1 (10) 1 |  |  |  |
| Urinary bladder hemorrhage |  | 1 (5) 1 |  |  |
| **Respiratory, thoracic, and mediastinal disorders** | | | | |
| Respiratory distress |  | 2 (11) 2 |  | 1 (5) 1 |
| Acute respiratory distress syndrome | 1 (10) 1 |  |  | 1 (5) 1 |
| Atelectasis |  |  |  | 1 (5) 1 |
| Hypercapnia |  |  |  | 1 (5) 1 |
| Pneumothorax |  | 1 (5) 1 |  |  |
| **Vascular disorders** | | | | |
| Peripheral ischemia |  | 1 (5) 1 |  |  |

**Additional file 1: Figure S1** **Mean arterial pressure over time.** All patients with a reported value are included, also if being out of shock (i.e. without any vasopressor support). Data points are means, error bars are omitted in order to improve readability.

**Additional file 1: Figure S2. Proportion of patients out of shock during the first 72 hours of treatment with selepressin or placebo.** Out of shock is defined as alive and off all vasopressors, including selepressin. Error bars represent SE.

**Additional file 1: Figure S3. Kaplan-Mayer estimation of time to septic shock resolution.** Septic shock resolution is defined as off all vasopressors with MAP ≥60 mmHg.

**Additional file 1: Figure S4. Proportion of days alive and off ventilator during the 7-day period from start of selepressin/placebo treatment.** Results are presented as means with SE.

The difference between selepressin 2.5 ng/kg.min and placebo is significant (p<0.02).

**Additional file 1: Figure S5.** **Mean cumulative urine output over time in septic shock patients.** All patients with a reported value are included. Error bars are omitted to improve readability.

**Additional file 1: Figure S6.** **Mean lactate over time in septic shock patients.** All patients with a reported value are included. Bars indicate SE.

**Additional file 1: Figure S7.** **Mean creatinine over time in septic shock patients.** All patients with a reported value are included. Bars indicate SE.
